# Three-dimensional array of microbubbles sonoporation of cells in microfluidics

**DOI:** 10.3389/fbioe.2024.1353333

**Published:** 2024-02-14

**Authors:** Guangyong Huang, Lin Lin, Quanhui Liu, Shixiong Wu, Jiapeng Chen, Rongxing Zhu, Hui You, Cuimin Sun

**Affiliations:** ^1^ School of Mechanical Engineering, Guangxi University, Nanning, China; ^2^ School of Mechanical and Automotive Engineering, Guangxi University of Science and Technology, Liuzhou, China; ^3^ Animal Science and Technology College, Guangxi University, Nanning, China; ^4^ School of Computer, Electronics and Information, Guangxi University, Nanning, China

**Keywords:** sonoporation, acoustic streaming, acoustic radiation force, membrane disruption, microfluidics

## Abstract

Sonoporation is a popular membrane disruption technique widely applicable in various fields, including cell therapy, drug delivery, and biomanufacturing. In recent years, there has been significant progress in achieving controlled, high-viability, and high-efficiency cell sonoporation in microfluidics. If the microchannels are too small, especially when scaled down to the cellular level, it still remains a challenge to overcome microchannel clogging, and low throughput. Here, we presented a microfluidic device capable of modulating membrane permeability through oscillating three-dimensional array of microbubbles. Simulations were performed to analyze the effective range of action of the oscillating microbubbles to obtain the optimal microchannel size. Utilizing a high-precision light curing 3D printer to fabricate uniformly sized microstructures in a one-step on both the side walls and the top surface for the generation of microbubbles. These microbubbles oscillated with nearly identical amplitudes and frequencies, ensuring efficient and stable sonoporation within the system. Cells were captured and trapped on the bubble surface by the acoustic streaming and secondary acoustic radiation forces induced by the oscillating microbubbles. At a driving voltage of 30 Vpp, the sonoporation efficiency of cells reached 93.9% ± 2.4%.

## 1 Introduction

The safe and efficient intracellular delivery of biologically active macromolecules into living cells is a challenging and critical process for research and therapeutic purposes in biotechnology Sharei et al. (2013); Liu et al. (2019); Sharei et al. (2014); Yoon et al. (2016, 2017). Intracellular delivery is a critical step in biological research and is applied in fields such as disease treatment and biomanufacturing. Methods for delivering exogenous cargoes can be categorized into biochemical and physical methods. Commonly employed biochemical methods include viral vectors and cationic lipids. Viral vector methods are associated with cytotoxicity, while cationic lipids do not offer uniform and dosage-controlled delivery across within a cell population Lundstrom (2018); Carugo et al. (2015); Stewart et al. (2018). Physical methods primarily involve transiently perforating the cell membrane using acoustic, optical, electrical, and mechanical methods to facilitate the intracellular delivery of exogenous cargoes Morshedi Rad et al. (2021); Chakrabarty et al. (2022); Rasouli et al. (2023); Wang and Lu (2006); Uvizl et al. (2021). Of the physical permeabilization approaches, sonoporation wields great potential and has been demonstrated as an efficacious technology for delivering a variety of functional cargos to different types of cells Azmin et al. (2012); Wu et al. (2006); Rich et al. (2022).

Sonoporation involves the disrupting of the cell membrane in the presence of microbubbles created through acoustic cavitation. Currently, most studies on ultrasound cavitation effects have been conducted at a macroscopic scale, where microbubble sizes exhibit a wide distribution, their positions are randomly distributed, and the distance between microbubbles and cells is uncontrollable Zhou et al. (2012); Qin et al. (2016). These factors directly impact delivery efficiency. In recent years, with the development of Micro-Electro-Mechanical Systems (MEMS) technology, microfluidic technology has emerged as a solution. Microfluidic technology has the advantages of low sample consumption, simple operation, multi-functional integration, small size and easy portability Whitesides (2006); Schaerli et al. (2009). The generation of microbubbles within microfluidic channels can be achieved using active methods such as electrical, optical, acoustic, thermal, mechanical and magnetic, *etc.* Gao et al. (2020). A commonly used method for generating microbubbles in microfluidic devices is to place blind-side pits on the walls of microchannels Patel et al. (2012). When the main microchannel is filled with liquid, air becomes trapped in these side pits, forming microbubbles with a consistent diameter Volk and Kähler (2018). These microbubbles can act individually or collectively to manipulate cellular behavior by regulating microscale flows Liu J. et al. (2019).

Currently, there is a growing trend in combining microbubbles and ultrasound within microfluidics for the study of sonoporation of cells. Due to the inverse relationship between the shear force induced by microbubbles and their diameter Meng et al. (2019), the diameter of microbubbles cannot be too large and typically ranges in the order of tens of micrometers. Moreover, research by Marin et al. has shown that the effective distance for microbubble action is approximately 3.25-fold their diameter Marin et al. (2015). Consequently, microchannel dimensions in microfluidics are often designed to be relatively small. When compared to other methods of cell perforation, the combination of sonoporation with novel microfluidic techniques offers certain advantages. Microinjection in microchannels enables precise cell puncturing, but it has lower throughput Adamo and Jensen (2008); Ghaemi et al. (2017). Optoporation allows for precise localization of perforation sites but involves expensive laser equipment and limited throughput Yuan et al. (2015); Li et al. (2013); Fan et al. (2015). In microfluidics cell squeezing techniques, there is a risk of microchannel clogging due to the comparable size of microchannels and cells Salari et al. (2021). However, in microfluidics sonoporation, challenges related to microchannel clogging and low throughput still need to be overcome. Our experiments also found that excessively small microchannel dimensions can lead to a low number of cells entering, further affecting the efficiency of sonoporation. Gac et al. employed a single cavitation microbubble for sonoporation on suspended cells, with the number of cells processed ultrasonically being less than 100 Le Gac et al. (2007). Meng et al. used microbubbles trapped in microcavities for stable cavitation to enhance the membrane permeability of individual cells Meng et al. (2019). Although using parallel arrays of microbubbles can increase the number of cells subjected to ultrasound treatment, overly small microchannels (height:50 *μ*m, width:240 *μ*m) hinder cell entry and are prone to clogging. It was reported that cavitation phenomena in small microchannels are generally weaker than that in larger microchannels under the same ultrasound field Dong et al. (2020). Small microchannels greatly restrict microbubbles from modulating cell membrane permeability.

There are many advantages in microfluidics sonoporation, but these improvements are needed in order to promote practical applications: i) Achieving high-efficiency and stable sonoporation while maintaining cell viability. ii) Flexibility in throughput to accommodate both the investigation of single-cell sonoporation mechanisms and research on cell delivery with a specific throughput. To address these issues, we propose a microfluidic chip that distributes three-dimensional array of microbubbles within microchannels, allowing for ultrasound treatment of cell clusters. Specifically, microbubbles are positioned on the sidewalls and the top of the microchannels, and they oscillate collectively under the influence of ultrasound within the microchannel. Finite element simulations optimize the structural dimensions of the microchannels by analyzing the acoustic streaming generated by oscillating microbubbles. When a fluid is injected into the microchannels using a syringe pump, surface tension forces produce three-dimensional array of microbubbles with consistent diameters. Acoustic streaming induced by oscillating microbubbles propels cells toward the microbubbles, while secondary acoustic radiation forces trap cell clusters at the bubble surfaces. Under the influence of shear forces, cell membrane deformation occurs, altering membrane permeability. Experimental results indicate that at a driving voltage of 30 Vpp, the sonoporation efficiency for cell clusters reaches 93.9% ± 2.4%. In a 14 mm microchannel, 5,115 cells are simultaneously processed using ultrasound. This device is not only suitable for single-cell-level research but can also significantly increase cell throughput by increasing the microchannel length or utilizing parallel microchannels.

## 2 Simulations and experimental

### 2.1 Microfluidic chip preparation

We designed and prepared a microfluidic chip based on cured polydimethylsiloxane (PDMS) for cell sonoporation experiments (Supplementary Figure S1†). Firstly, the structural dimensions of the microchannels in the microfluidic chip were determined. An effort was made to ensure that the influence of microbubbles was experienced by each cell in spatial terms, as the effective distance of microbubbles influence is approximately 3.25-fold its diameter Marin et al. (2015). Simultaneously, the design of the top microbubbles and the two side wall microbubbles are basically the same distance from the centre of the microchannel. The designed microbubble diameter was 65 *μ*m. Considering the effective range of microbubble influence and retaining a certain amount of reserve, the initial dimensions of the 14 mm long microchannel cross-section were determined to be 800 *μ*m in width and 200 *μ*m in height. Next, the fabrication method for the chip was determined. Due to the relatively complex structure of the microchannel in the chip, using photolithography techniques would require multiple exposure processes. In order to reduce costs and complexity, we used 3D printing machine (NanoArch P140, BMF Precision, China) for one-step fabrication. We used photosensitive resin materials Yellow-20 (BMF Precision, China). The resolution of the 3D printer device is 10 *μ*m.

**FIGURE 1:**
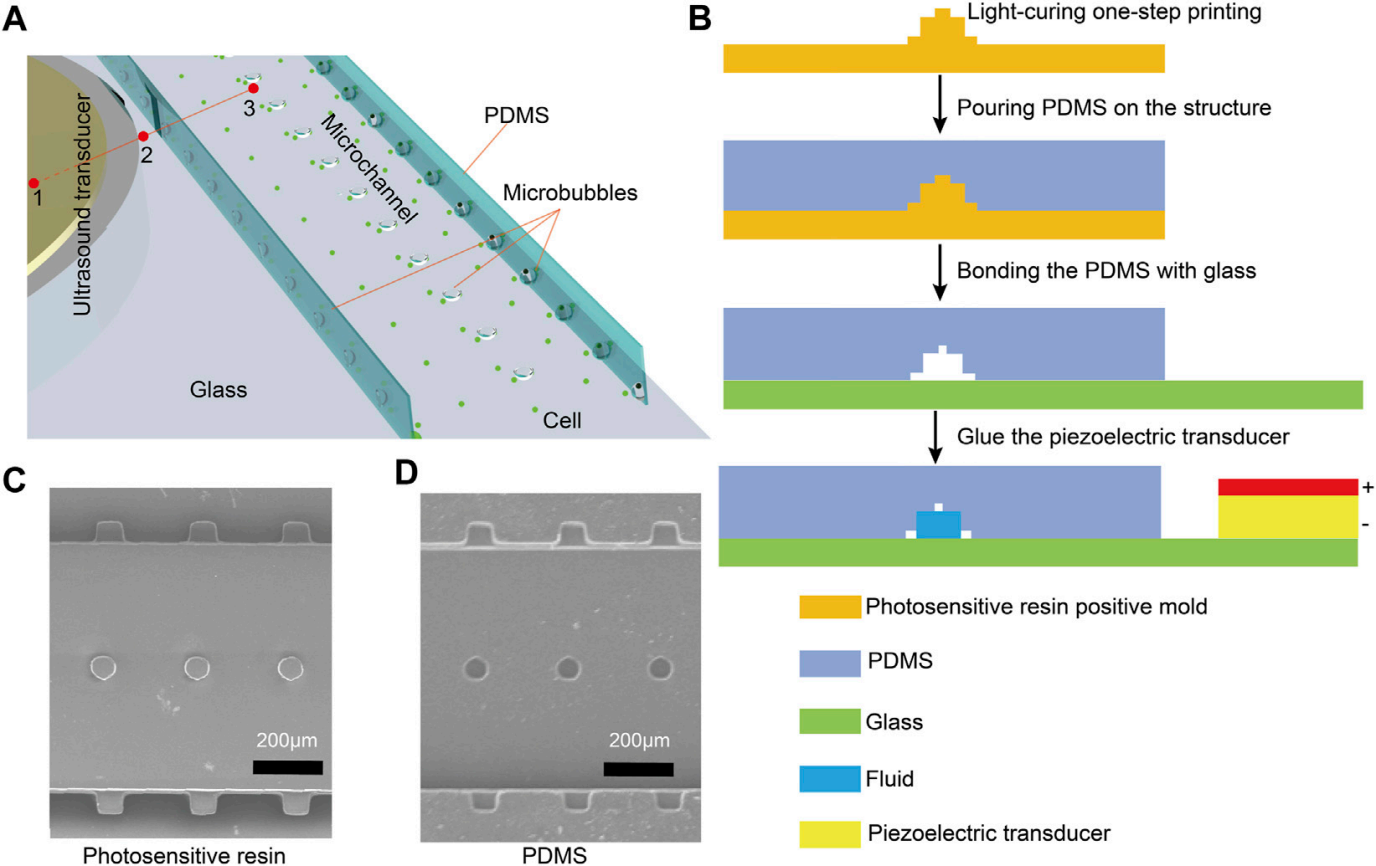
**(A)**Schematic diagram of the microfluidic chip device. The marks 1, 2, and 3 represent the three positions for temperature measurement. **(B)** The fabrication process of the microfluidic chip is based on photoresin printing and PDMS molding. The ultrasound transducer is affixed to the glass using adhesive. **(C)** Top view of the photosensitive resin chip structure taken by Scanning Electron Microscope. **(D)** Scanning Electron Microscope photograph of the structure of PDMS torn from the photosensitive resin.

**FIGURE 2:**
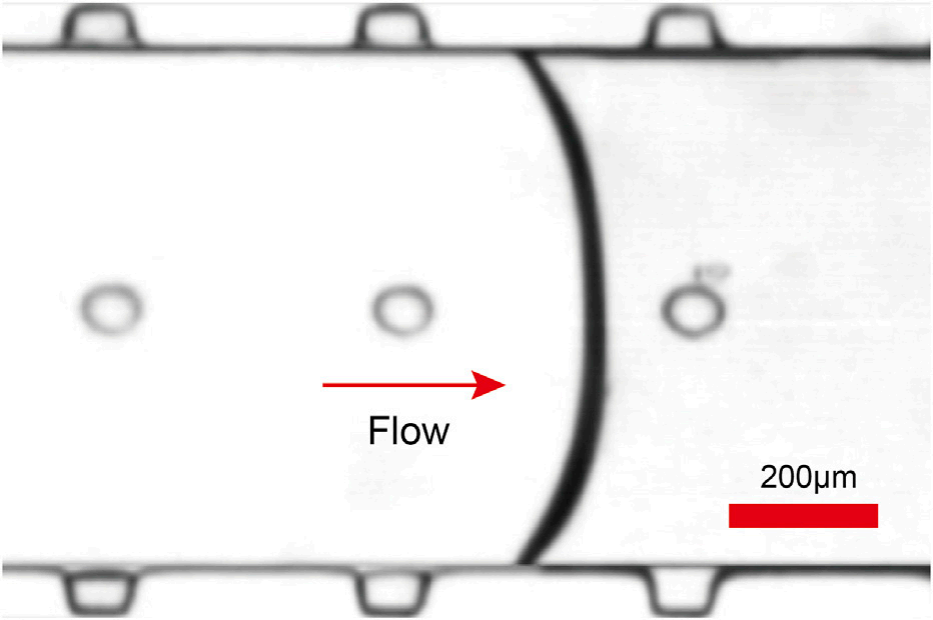
As the fluid flows through the microchannels, microbubbles of the same diameter are generated in the microcavities at the side walls and top due to surface tension.

In this paper, a microfluidic chip made of PDMS was designed, which consists of a flow channel for cellular solutions with an array of microcavity structures on both sides and top (Figure 1A). The fabrication process of the microfluidic chip is based on photoresin printing and PDMS molding (Figure 1B). Firstly, a 3D high-resolution photosensitive resin printer was utilized to print the chip mold. To ensure better mold formation and casting, the mold was soaked in alcohol for 1.5 h, then air-dried and baked in an oven for 8 h. PDMS prepolymer and curing agent (Sylgard 184, Dow Corning, United States) were poured onto the photosensitive resin template in a 10:1 mixture. The mixture was degassed for 10 min in a vacuum chamber (DZF6090, JingHong, China), followed by a 6 h settling on a level platform. Subsequently, it was cured for 2 h at 60°C, and the PDMS was peeled off. Inlets and outlets for microchannels were manually created. The peeled PDMS and the photosensitive resin chip mold were examined under a Scanning Electron Microscope (EVO100, Zeiss, Germany) to observe the structure (Figures 1C, D). From the images, it is evident that the PDMS faithfully replicated the mold’s structure. The surface roughness Ra of the resin mold was measured to be 38.83 nm using probe-type surface profiler (DektakXT, Bruker) ((Supplementary Figure S2†). Subsequently, PDMS and glass were subjected to a 60-s oxygen plasma treatment in a mid-range plasma cleaner (PDC-002, HARRICK, United States) to enable their permanent bonding. Finally, the ultrasound transducer was coupled with epoxy resin (deli, China) and thin glass (guluo, China) (length: 5 mm, width: 2.5 mm, height: 330 *μ*m), completing the fabrication of the PDMS chip. In order to generate and prolong the stable time of microbubbles, the fabricated chip was left for 24 h to provide sufficient time for the PDMS to regain its hydrophobicity. When the solution was injected into the chip at a flow rate of 5 *μ*L min^−1^, microbubbles were formed in the microcavities of the microchannel due to the influence of surface tension (Figure 2, Supplementary Movie S1 in the ESI†). Since the microcavities in the PDMS channels had identical dimensions, the array of microbubbles generated in the microfluidic chip maintained approximate uniformity in size. The microbubbles appear highly monodispersed (mean value 65.3 *μ*m) with a standard deviation of 2.4%.

### 2.2 Simulation three-dimensional array of microbubble acoustic streaming

**FIGURE 3:**
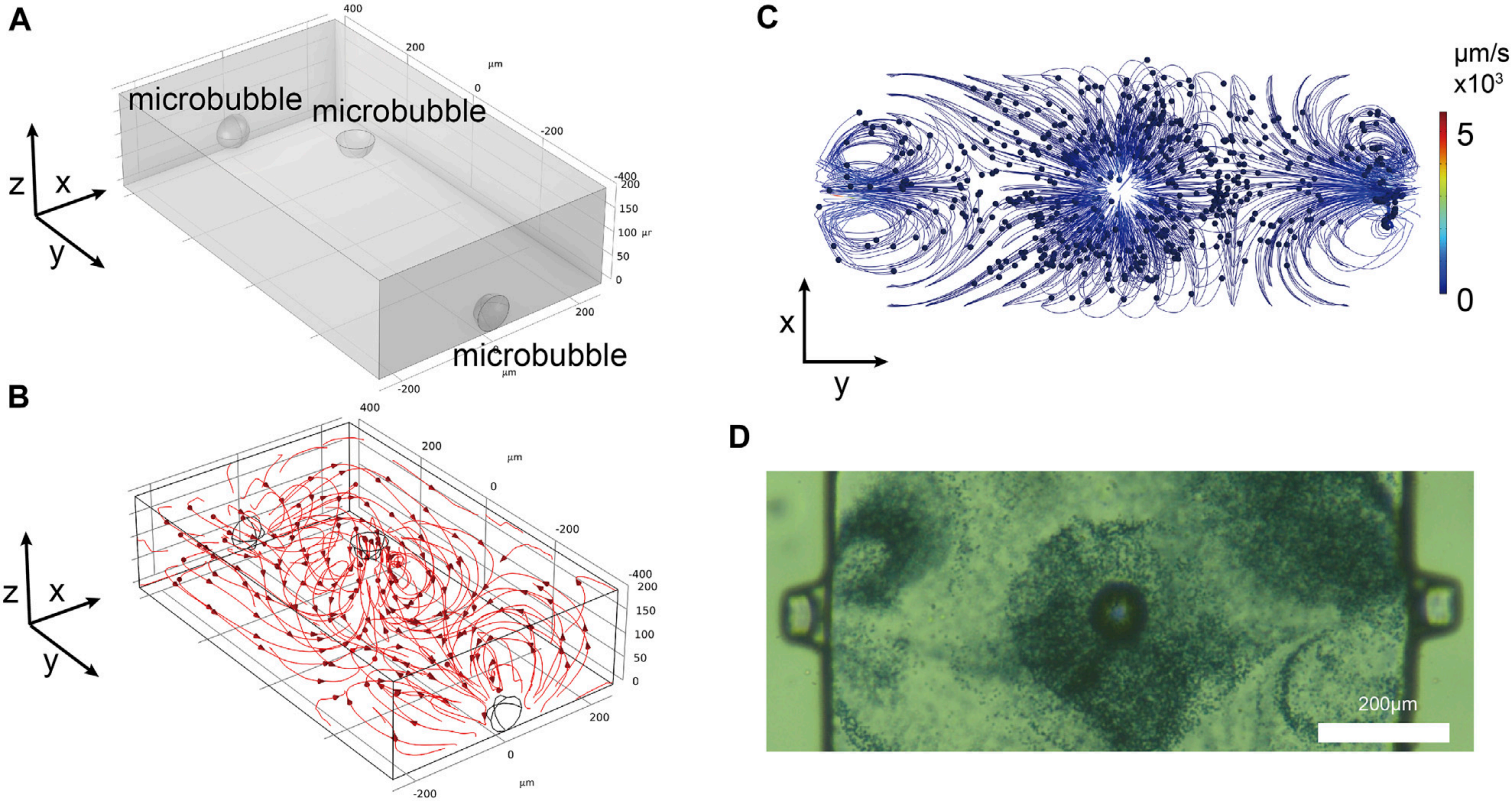
**(A)** Three-dimensional simulation model. **(B)** Streamlines formed by the simulated microbubbles. **(C)** Top-down view of particles movement in the simulation model. **(D)** Experimental observation of polystyrene particles movement around the microbubbles.

To harness the optimal effects of the microbubbles on all three surfaces of the microchannel, three-dimensional array of microbubbles acoustic streaming model was established (length: 500 *μ*m, width: 800 *μ*m, height: 200 *μ*m) (Figure 3A).

We simplified the model by considering only the microbubble cap and the liquid between them. Due to factors related to the chip fabrication process, the microbubbles on the side walls of the microchannel had to be positioned beneath the lateral surfaces. Owing to the nonlinear terms present in the Navier-Stokes equations, harmonic perturbations induced by external acoustic fields result in a net time-averaged fluid flow within the microchannel, which is referred to as acoustic streaming. In this research, our focus is primarily on the time-averaged equations. The time average over the entire oscillation period is represented as ⟨ ⟩, and the second-order continuity equation and the Navier-Stokes equation are as follows Muller et al. (2012).

$$\rho_{0}\nabla \cdot \left\langle v_{2}\right\rangle =-\nabla \cdot \left\langle \rho_{1}v_{1}\right\rangle$$ (1)

$$\eta \nabla^{2}\left\langle v_{2}\right\rangle +\beta \eta \nabla \left(\nabla \cdot \left\langle v_{2}\right\rangle \right)-\left\langle \nabla p_{2}\right\rangle =\left\langle \rho_{1}\partial_{t}v_{1}\right\rangle +\rho_{0}\left\langle \left(v_{1}\cdot \nabla \right)v_{1}\right\rangle$$ (2)

where *ρ*_0_ is density, *ρ*_1_ is first order density, ***v***_1_ is first order velocity, ***v***_2_ is second order velocity, *η* is dynamic shear viscosity, *β*is viscosity ratio, *p*_2_ is second order pressure.

The streamline diagram of ultrasound-induced acoustic streaming is shown in Figure 3B. The streamlines are all centered around the microbubbles, forming vortices that flow towards the vertex. In experiments, it is common to use particles to simulate the flow patterns of acoustic streaming. In this study, the movement of 2 *μ*m diameter polystyrene tracer particles was employed as a tracking fluid. A simulation model was also developed to simulate the interaction of acoustic streaming with the motion of these polystyrene particles. The standard expression defines the time-averaged Stokes drag force experienced by a spherical particle with a radius *r* that is moving with velocity ***u*** in a fluid characterized by a streaming velocity $\left\langle v_{2}\right\rangle$ Muller et al. (2012).

$$F^{\text{drag}}=6\pi \eta r\left(\left\langle v_{2}\right\rangle -u\right)$$ (3)

Additionally, the Particle Tracing for Fluid Flow module of COMSOL software was used to simulate the motion of 2 *μ*m diameter polystyrene particles. Simulations were conducted with a diameter of 2 *μ*m polystyrene particles evenly distributed within the microchannel. When viewed from top, the polystyrene particles were predominantly influenced by the three microbubbles (Figure 3C, Supplementary Movie S2 in the ESI†). In the axonometric view, the polystyrene particles with a diameter of 2 *μ*m were observed to move around each microbubble. When observed experimentally from a top view (Figure 3D), the direction and distribution of the polystyrene particles movement matched the simulation results. The polystyrene particles congregated on the top of the microbubble caps and along the sidewalls in an anti-fountain manner. To gain a better understanding of the extent of the influence of the microbubbles, the motion of polystyrene particles was observed from an axonometric angle (Supplementary Movie S3 in the ESI†). From the simulation results, it was evident that the microbubbles effectively influenced nearby particles, in accordance with the anticipated outcomes. The other simulation conditions were the same, except that the top microbubbles were reduced and the particles away from the microbubbles were essentially immobile (Supplementary Movie S4 in the ESI†).

### 2.3 Three-dimensional array of microbubbles sonoporation mechanisms

**FIGURE 4:**
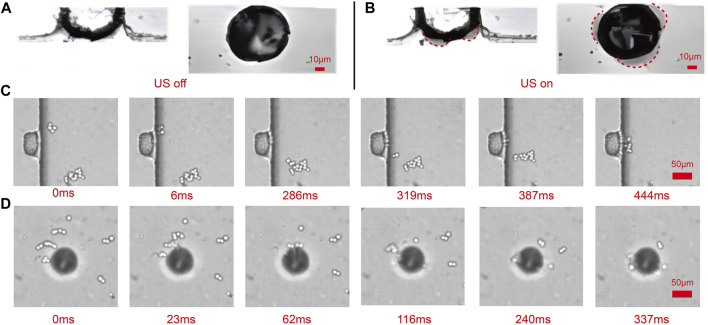
Observation of the shape of sidewall and top microbubbles and trapped captured cells based on the high-speed camera and the microscope. **(A)** The shape of the microbubble in the absence of ultrasound presence. **(B)** The shape of the microbubble in the presence of ultrasound. **(C)** With the presence of ultrasound, all cells in the microchannel are attracted toward the oscillating sidewall microbubbles in 444 ms. **(D)** With the presence of ultrasound, all cells in the microchannel are attracted toward the oscillating top microbubbles in 337 ms.

Using a combination of a high-speed camera and a microscope, the deformation of the side and top microbubbles was observed (Figures 4A, B). During ultrasound exposure, both the top and side microbubbles exhibited significant deformation. The red dashed lines in the figure represent the contour of microbubble deformation, indicating that the microbubbles oscillate on their surfaces. The resonance frequency of the microbubbles can be experimentally determined by examining the response of the maximum oscillation amplitude to the applied frequency. Under the influence of ultrasound, the surface of the microbubbles exhibits intense oscillation, forming capillary waves on the bubble surface (Supplementary Movie S5 in the ESI†). The resonance frequency of a trapped microbubble in stationary fluid is estimated by the Rayleigh - Plesset equation Birkin et al. (2011).

$$f_{\text{t}}=\frac{1}{2\pi R_{0}\sqrt{\rho }}\sqrt{3k\left(p+\frac{2\sigma }{R_{0}}\right)-\frac{2\sigma }{R_{0}}}$$ (4)

where *ρ* is the density of the Phosphate Buffer Saline (PBS) solution, *σ* is surface tension of the PBS solution, and *k* is the polytropic exponent for a bubble containing air, *p* is the static pressure, and *R*_0_ is the radius of the microbubble.

**FIGURE 5:**
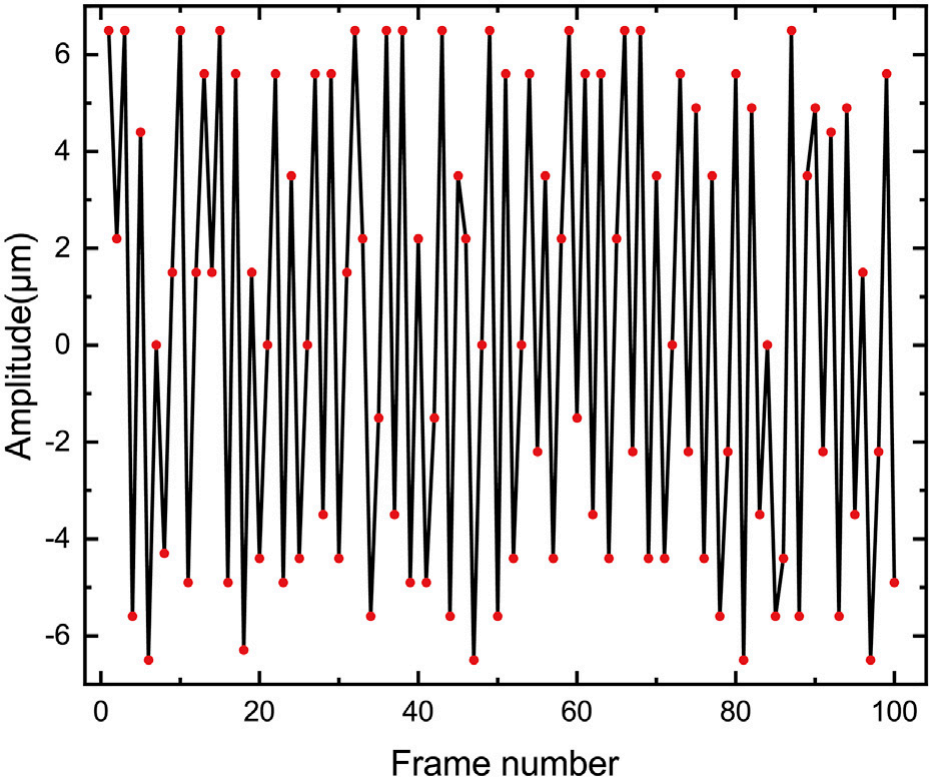
The variation of the amplitude of the oscillating microbubbles with the number of frames at 97.5 kHz and 30 Vpp was recorded using a stroboscopic technique.

According to Eq. 4, the resonance frequency of microbubbles is calculated as 95.1 kHz (see Supplementary Note S1†). This value is approximately the ultrasound frequency, which is 97.5 kHz. Even though the high-speed camera sampling rate (80 kHz) is less than the microbubble resonance frequency (95.1 kHz), the very short exposure time and carefully chosen driving frequencies allow us to improve the time resolution using stroboscopic techniques Wang et al. (2013). We utilized stroboscopic techniques, cleverly selecting the sampling frequency, to capture 100 images of the same microbubble at different times and measure the amplitude. This allowed us to estimate the amplitude of the bubble’s oscillation. The maximum amplitude of the bubble’s oscillation is approximately 6.5 *μ*m (Figure 5).

Oscillating sharp-edge structures or microstreaming direct current velocities drop rapidly on viscous boundary layers near microbubbles. When cells are situated in this vicinity, the primary cause of cell damage is the substantial shear stress resulting from the velocity gradient. When cells are trapped in this region, shear stress near the oscillating sharp-edge structures or microbubbles is estimated using equation Wu (2007).

$$S=2\pi^{3/2}\varepsilon^{2}{\left(\rho f_{t}^{3}\mu \right)}^{1/2}/R_{0}$$ (5)

where *ρ* is the density of the PBS solution, *ɛ* is the oscillating amplitude of the microbubble, *μ* is the dynamic viscosity of the PBS solution, *f*_*t*_ is oscillation frequency, *R*_0_ is the radius of the microbubble.

The shear stress threshold for cell sonoporation is 12 ± 4 Pa Wu (2007). From the experiment, the amplitude of the microbubble was measured as 6.5 *μ*m, and according to Eq. 5, it can be determined that the shear stress near the microbubble is 455 Pa (see Supplementary Note S2†). This shear force significantly exceeds the threshold, which indicates that it is sufficient to achieve cell sonoporation. For the case of a moving cell rather than a stationary point near an oscillating microbubble, Eq. 5 can be used to estimate the upper bound of shear stress.

The trapping mechanism can be explained by two main forces acting on cells due to the oscillating bubble. These forces consist of the drag force induced by microstreaming and the secondary acoustic radiation force generated by the oscillations of the bubble. The secondary radiation force, originates from the pulsating bubble and can be estimated by Shin et al. (2017).

$$F_{\text{r}}=4\pi \frac{\rho -\rho_{\text{p}}}{\rho +2\rho_{\text{p}}}\frac{R_{0}^{4}R_{\text{c}}^{3}}{d^{5}}\omega^{2}\varepsilon^{2}$$ (6)

where *R*_0_ is the radius of a microbubble, *R*_*c*_ is the radius of the cell, *d* is the distance between the centers of the microbubble and the cell, *ω* is the angular oscillation frequency, *ϵ* is the oscillating amplitude of the microbubble, and *ρ* and *ρ*_*p*_ are the densities of the PBS solution and the particle, respectively.

From Eq. 6, it is evident that the direction of the secondary radiation force is determined by the relative density of the surrounding fluid and cellsHashmi et al. (2012). Cells having a higher density (*ρ* > *ρ*_*p*_) are drawn toward the oscillating bubbles, while cells with a density lower than that of the surrounding medium are pushed away (*ρ* < *ρ*_*p*_). In the experiment, the cells had slightly higher density than the liquid and were attracted to the oscillating bubbles (Figures 4C, D). During the bubble’s oscillation, the attracted cells remained trapped on the bubble’s surface. Almost all cells suspended in the microchannel are trapped and the cell trapping rate reached 93.4% ± 1.96%.

### 2.4 Effect of temperature on cells

**FIGURE 6:**
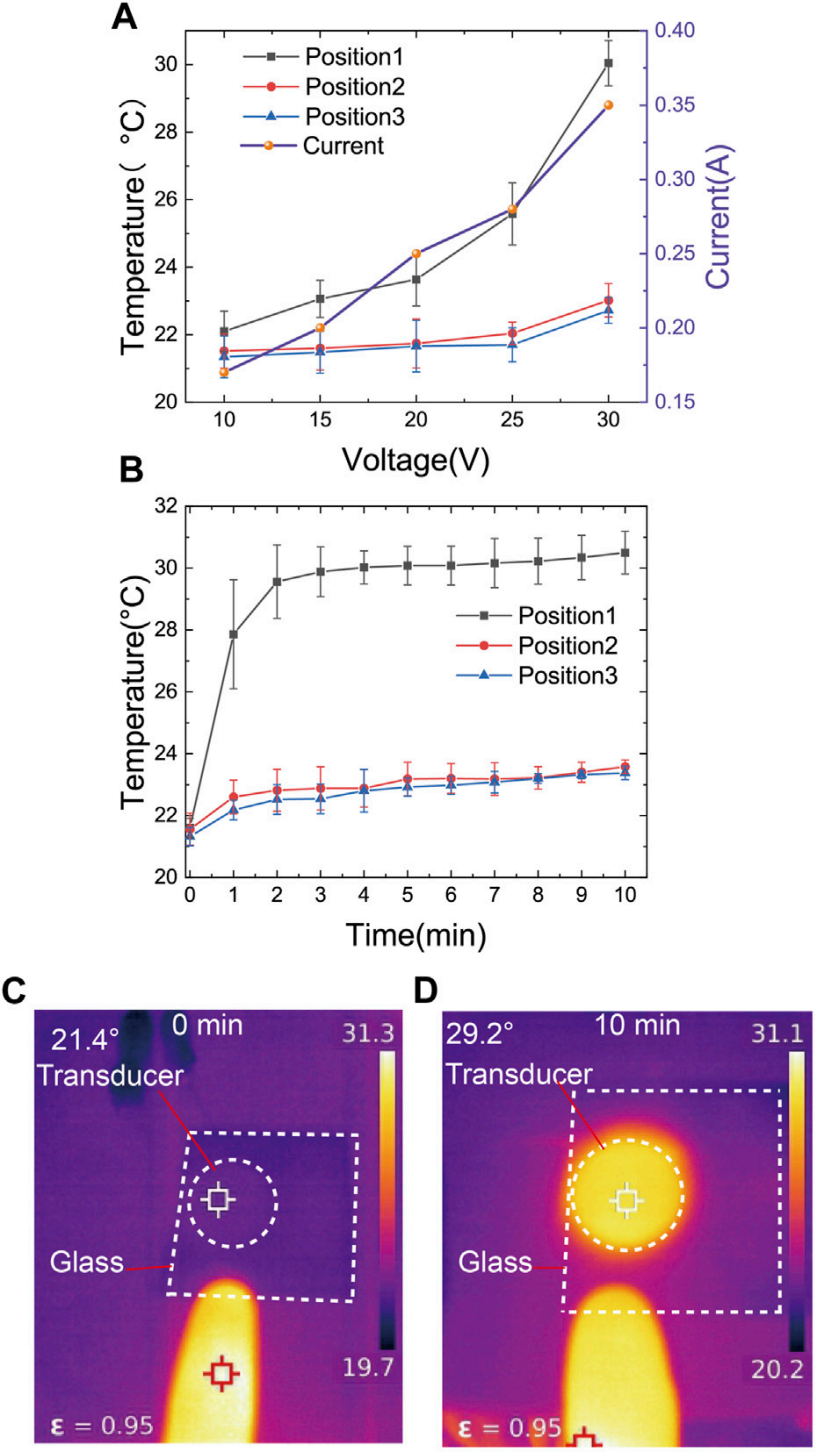
**(A)**The temperature of the substrate was measured with a thermal imaging camera in three regions (Marked as 1,2,3 in Figure 1, glass placed on the top of the PZT) at the ultrasound transducer frequency of 97.5 kHz, with driving voltages of 10 Vpp, 15 Vpp, 20 Vpp, 25 Vpp, and 30 Vpp for 10 min of operation, respectively. The values of current flowing through the transducer corresponding to driving voltages of 10 Vpp, 15 Vpp, 20 Vpp, 25 Vpp, and 30 Vpp, respectively, were also recorded. **(B)** Measurement of substrate temperature changes in three regions at 1-min intervals at an ultrasound transducer frequency of 97.5 kHz and a drive voltage of 30 Vpp. **(C,D)** Temperature comparison between a human finger and the transducer during acoustic operation.

Before experiments, it was necessary to account for the temperature influence resulting from the main heat source in the system, which is the electromechanical losses in the ultrasound transducer. The substrate temperature was measured using a temperature measuring instrument (botterrun rx-680, A-BF, China) in a constant temperature environment in a clean room. Temperature measurements were taken at three positions: the center of the ultrasound transducer (position 1), the edge of the ultrasound transducer (position 2), and the center of the microchannels (position 3) (Figure 1A). The glass substrate temperatures were measured with the ultrasound transducer operated at a frequency of 97.5 kHz and driven at various voltages (10 Vpp, 15 Vpp, 20 Vpp, 25 Vpp, 30 Vpp) for 10 min. As the voltage increased, the temperatures at the three positions also increased (Figure 6A).

When the ultrasound transducer was driven at 30Vpp, the temperatures at all three positions remained relatively stable after 10 min of operation (Figure 6B). From the results, it can be observed that when the ultrasound transducer was operated at a frequency of 97.5 kHz and driven at 30 Vpp for 10 min, the highest temperature recorded at the center of the transducer was only 29.2°C (Figure 6C). This temperature remains lower than normal body temperature, and the heat generated has minimal impact on cell damage.

## 3 Results and discussion

**FIGURE 7:**
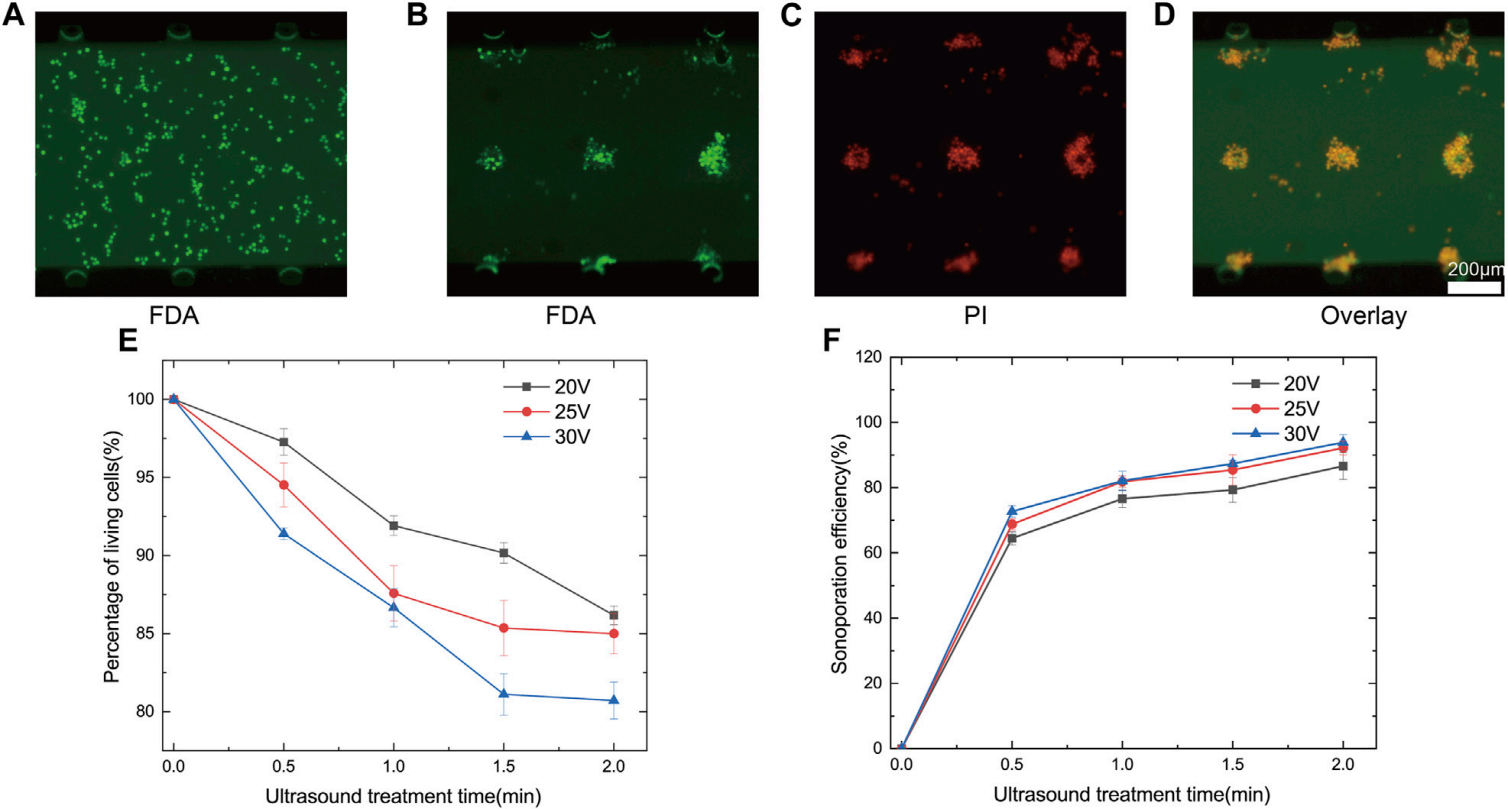
**(A)** Observation of the state of cells unaffected by ultrasound, emitting green fluorescence under FDA staining reagent, the cells were viable and evenly distributed in the microchannel. **(B)** Three-dimensional array of microbubbles oscillated traps nearby cells around the microbubbles and emitted green fluorescence under FDA staining reagent, indicating that the cells were still viable. **(C)** Cell clusters in the action of three-dimensional array of microbubbles oscillated, emitting red fluorescence under PI stain. **(D)** The merged fluorescence images show that three-dimensional array of microbubbles oscillation allows the cell clusters to obtain high sonoporation efficiency. **(E)** Percentage of living cells as a function of ultrasound exposure time at different voltages. **(F)** Sonoporation efficiency as a function of ultrasound exposure time at different voltages.

To validate the high efficiency of ultrasound treatment using three-dimensional array of microbubbles, multiple cavities with identical structures were designed. Human renal epithelial (293T) cell lines present in this study were obtained from Cell Bank, Chinese Academy of Sciences (Shanghai, China). The cell solution was injected into the microchannel at a flow rate of 5 *μ*L min^−1^ using a high-precision syringe pump. Due to the influence of surface tension, microbubbles were formed within the microcavities. Since the dimensions of the microcavities, produced by high-precision 3D printing, were nearly identical, uniformly sized microbubbles were created. The suspended 293T cells were evenly distributed within the microchannel (Figure 7A).

When the ultrasound transducer is driven by the power amplifier, the sound wave propagates along the glass substrate and oscillates, which causes the microbubbles inside the microchannel to oscillate. Oscillating microbubbles captured and adhered to nearby 293T cells. Due to the optimized layout of three-dimensional array of microbubbles, cells in all spatial regions were effectively affected. The captured cells rotated and clustered around the microbubbles (Supplementary Movie S6 in the ESI†). Due to the presence of a substantial number of cell clusters, a brief pause during ultrasound exposure was introduced for improved sonoporation. This pause allowed cells to reassemble and aggregate after dispersing in the absence of ultrasound, ensuring that all cells were uniformly exposed to the shear forces from the microbubbles (Supplementary Movie S7 in the ESI†). Cell activity and membrane permeability were assessed using FDA/PI staining. Each cell cluster captured around a microbubble emitted green fluorescence (Figure 7B), while overall the permeability of cell clusters was enhanced (Figure 7C). Overlapping fluorescence images of FDA/PI indicated that cell clusters were effectively sonoporated (Figure 7D). We analysed percentage of living cells and sonoporation efficiency over time at different driving voltages. As voltage and ultrasound exposure time increased, percentage of living cells decreased. When the voltage was set at 30Vpp and ultrasound exposure time increased from 0.5 min to 2 min, percentage of living cells decreased from 91.4% ± 0.4% to 80.7% ± 1.9%. However, with increased driving voltage and exposure time, sonoporation efficiency improved (Figure 7F). With a voltage of 30Vpp and 2 min of ultrasound exposure, cell sonoporation efficiency reached 93.9% ± 2.4%. Percentage of living cells and sonoporation efficiency were both related to voltage and ultrasound exposure time. Therefore, we should consider the sonoporation efficiency along with the percentage of living cells fully. With the increase of ultrasound treatment time, percentage of living cells and sonoporation efficiency tended to saturate the trend. The possible reason for this was due to the fact that the cells were acted with different effects at different distances from the microbubbles. Cells that were far away were not acted upon as effectively and the activity and sonoporation efficiency tended to be more saturate with time. Therefore, short pauses at intervals during the experiment to change the position of the cells were necessary to allow all cells to act more evenly. Due to the change of microbubbles over time, there may have been a few cells that were unable to be trapped by microbubbles. When the microbubbles stopped oscillating, because the fluid did not stop flowing immediately, a few cells moved with the fluid. Therefore, the cells that were far away from the microbubbles in the figure showed red fluorescence (Figure 7C).

**FIGURE 8:**
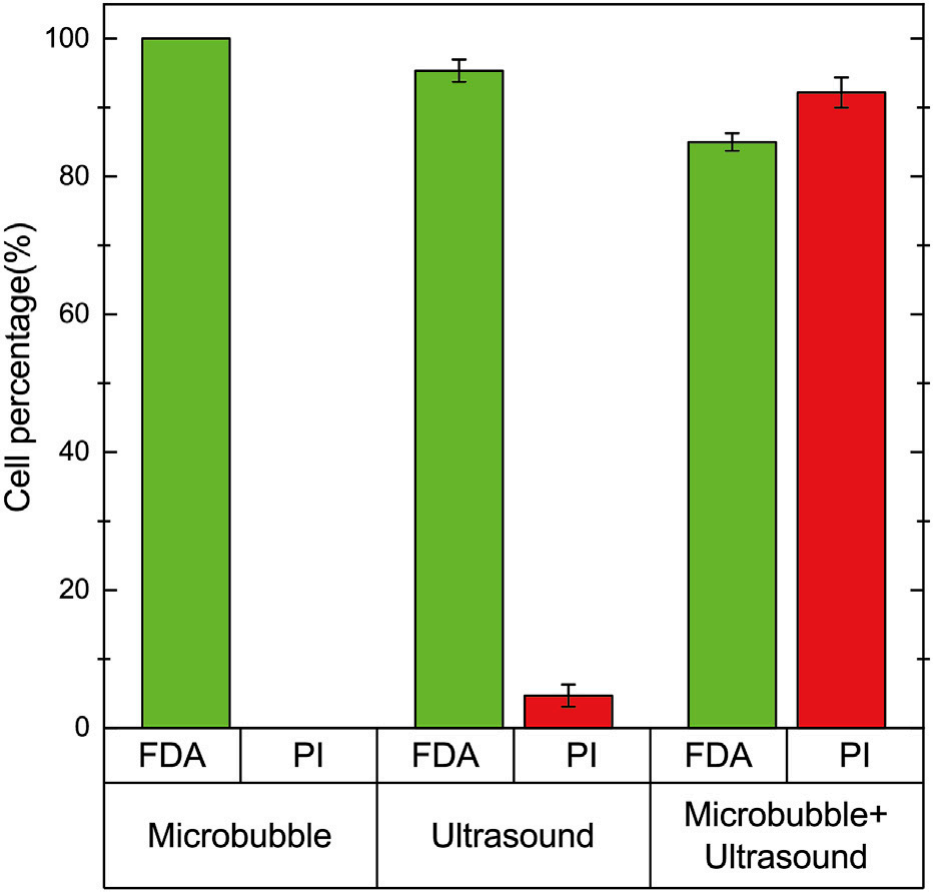
Cell staining experiments were performed on cells in the presence of microbubbles, ultrasound, and microbubble + ultrasound. When only microbubbles were present, all cells fluoresced green, and no red fluorescing cells were observed. When only ultrasound was used, 95.3 %± 1.6% of the cells fluoresced green and 4.7 %± 1.6% fluoresced red. When both ultrasound and microbubbles were present, 85.0 %± 1.3% of the cells fluoresced green and 92.2 %± 2.2% fluoresced red.

In order to demonstrate that ultrasound-driven microbubble oscillations correlate with cell activity and cell sonoporation, a comparative experiment was conducted with microbubbles, ultrasound, and ultrasound + microbubbles. The signal driving the ultrasonic transducer was still 25Vpp voltage and 97.5 kHz frequency. Only ultrasound without microbubbles was used in the comparative experiment, and the microchannel was designed with the original size, but without microcavities. The experiments were all conducted by introducing cell solutions into the microchannel and staining the cells after 2 min of the same time (Figure 8). When only microbubbles were used, there was no effect on cell activity, reaching 100%. When only ultrasound was used, a very small number of cells fluoresced red, but only accounted for 4.7 %± 1.6%. When microbubbles were driven by ultrasound oscillation, cells fluorescing green accounted for 85.0 %± 1.3%, and cells fluorescing red accounted for 92.2 %± 2.2%. Therefore, ultrasonic-driven microbubbles have a significant impact on cell activity and cell sonoporation.

Ultrasound-mediated microbubble oscillation is an efficient technique for cell sonoporation that finds applications in cell therapy, drug delivery, biomanufacturing, and more. With the advancement of microfabrication and lab-on-a-chip technologies, microbubble sonoporation within microfluidic chips offers a gentler and more controllable alternative to traditional macro-scale ultrasound, resulting in lower cell death rates. Increasingly, microbubble-based research in cell sonoporation is being conducted within microfluidic systems. Due to limitations in microbubble size and effective action range, the dimensions of microchannels designed for this purpose are typically small. While combining traditional physical methods with novel microfluidic approaches offers advantages, overcoming channel clogging and low throughput remains a challenge. These issues are typically associated with microfluidic techniques, especially when the precision of the method is scaled down to the single-cell level Salari et al. (2021); Deng et al. (2018). Very small microchannel dimensions can limit the number of cells that can be introduced, thus impacting sonoporation efficiency. In addition, under the same ultrasound field, small microchannels weaken the cavitation effect, resulting in the inability of cells to perforate Dong et al. (2020). Due to the limitations of the manufacturing chip process, the microcavities in the sidewalls are underneath the sides of the microchannels, thus limiting the height of the microchannels. By adding microbubbles at the top of the microchannel, enabling action from three directions on the cells, the microchannel’s height can be increased. As previously discussed, the microchannel height for the fabrication of three-dimensional array of microbubbles chip is 200 *μ*m. Traditional photolithography would require multiple exposures, whereas using a 3D high-precision machine allows for one-step printing.

Three-dimensional array of microbubbles can stably and efficiently facilitate cell sonoporation. Meng et al. have already demonstrated that ultrasound action on microbubble produces stable cavitation, which is more stable and controllable for sonoporation compared to inertial cavitation Meng et al. (2019). The designed microcavity structures maintain consistent dimensions, generating microbubbles of nearly identical size. Under the influence of the same ultrasound, these microbubbles oscillate with nearly the same amplitude. Moreover, the microbubble generated at the air/liquid interface without employing a shell is capable of oscillating freely, enhancing the efficiency of sonoporation. Recent studies on sonoporation in combination with the acoustic streaming approach suggest that acoustic streaming plays a large role in enhancing cell membrane permeability Salari et al. (2021); Zhao et al. (2021); Rasouli et al. (2023). Therefore, the acoustic streaming generated by the oscillation of three-dimensional array of microbubbles can capture cells and evenly mix them while enhancing cell membrane permeability (Supplementary Movie S7 in the ESI†). This is also the reason for the brief pausing during the ultrasound exposure, which allows for the re-mixing of captured cells and achieves better sonoporation results.

Three-dimensional array of microbubbles microbubble-based cell sonoporation method increases the number of cells. After the cell solution was injected in the 14 mm long microchannel, the green fluorescent cells are counted in three randomly selected fields of the same area, which are converted to 5,115 cells in the whole microchannel. Currently, other micro-nano techniques for cell membrane permeabilization Aghaamoo et al. (2022), such as microinjection, sharp-edge induced acoustic streaming, usually do not exceed 200 cells Zhao et al. (2021); Shekaramiz et al. (2016); Chow et al. (2016). Typically, traditional sonoporation of suspended cells is performed within a single channel, yielding fewer than 100 sonoporated cellsLe Gac et al. (2007). By increasing the microchannel length or parallelizing more microchannels, the efficiency of ultrasound treatment can be further enhanced. Therefore, this study has the flexibility of both single-cell, higher-throughput sonoporation.

## 4 Conclusion

The use of oscillating microbubbles in microfluidics to enhance cell membrane permeability is a highly promising biotechnological method. Compared to traditional macro-scale microbubble-assisted sonoporation, microbubble oscillation in microfluidics offers advantages of control, stability, efficiency, and high percentage of living cells. However, the current state of the art typically operates at single-cell or low-throughput levels, often in the range of hundreds of cells. In this study, we demonstrated a method for sonoporation within a microfluidic chip using three-dimensional array of microbubbles oscillation. By conducting simulation analyses to enlarge and optimize the chip’s dimensions, the chip can be one-step fabricated by a high-precision light curing 3D printer. The acoustic streaming generated by the oscillation of three-dimensional array of microbubbles captures and uniformly mixes trapped cells, enhancing cell membrane permeability. Under ultrasound conditions at a frequency of 97.5 kHz and a driving voltage of 30 Vpp, the sonoporation efficiency for cell clusters reached 93.9% ± 2.4%. Within a 14 mm microchannel, 5,115 cells can be simultaneously treated by ultrasound. By extending the microchannel length or parallelizing more microchannels, the device’s throughput can be further increased. Consequently, this research offers the flexibility to study single-cell-level sonoporation mechanisms and high-throughput intracellular delivery simultaneously.

## 5 Materials and methods

### 5.1 Cell culturing and particle preparation

The 293T cells were seeded in cell culture dishes containing complete culture medium Dulbecco’s Modified Eagle Medium (DMEM, Gibco, United States) with 10% fetal bovine serum (FBS, Gibco, United States) and placed in a humidified environment at 37°C with 5% *CO*_2_. After 2 days of cultivation, the cells were digested using 0.25% trypsin (Gibco, United States) and subsequently suspended in Phosphate Buffer Saline (PBS, Gibco, United States) containing penicillin-streptomycin (Gibco, United States). Finally, a cell solution with a concentration of approximately 10^6^ *m*L^−1^ was prepared and set aside. This cell solution was injected into the microchannels at a rate of 5 *μ*L min^−1^ using a syringe pump (Pump 11 Elite, Harvard Apparatus, United States). The microfluidic flow was observed by utilizing polystyrene particles (YUAN BIOTECH, China) with a diameter of 2 *μ*m. To prevent polystyrene particles from adhering to the bottom of the microchannels, the experiment employed 0.2% w/w Tween 20 (Biofroxx, Germany) as a surfactant.

### 5.2 Experimental equipment and data analysis

The experimental setup consists of a signal generator (33500B, KEYSIGHT, United States) and a voltage amplifier (ATA4-315, Aigtek, China), used to control the power and frequency of a 25 mm diameter PZT-4 ultrasound transducer. The experiments involve observing microbubble oscillations by sweeping through different frequencies. The maximum acoustic streaming caused by microbubble oscillations was observed at 97.5 kHz and thus the exciting frequency of 97.5 kHz was chosen for all the experiments. A high-speed camera (UX100, FASTCAM, Japan) connected to a upright microscope (DMi8, Leica, Germany) was used to capture the motion of cells in the acoustic microvortices. Acoustic streaming videos were recorded by the microscope (×10 eyepiece and ×10 objective) and the high-speed camera (500 frames s^−1^). The cell capture process was recorded by the microscope (×10 eyepiece and ×10 objective) and the high-speed camera (5,000 frames s^−1^). Microbubble deformations were recorded by the microscope (×10 eyepiece and ×20 objective) and the high-speed camera (80,000 frames s^−1^). All data are presented as mean ± SEM. Statistical analysis involved independent sample t-tests and analysis of variance for multiple group comparisons, using IBM SPSS 26 statistical software. A significance level of *p* < 0.05 was used to determine statistically significant differences.

### 5.3 Cell staining

Cell staining was performed by adding Propidium Iodide (PI, Solarbio, China) and fluorescein diacetate (FDA, yuanye, China) to the cell solution to assess the effectiveness of sonoporation. Following ultrasound exposure, if there were changes in cell membrane permeability, PI would penetrate the cell membrane and bind with DNA and RNA, emitting red fluorescence. At the same time, FDA only stained viable cells, producing green fluorescence. The concentrations of PI and FDA used in the experiment were 6 *μ*M and 20 *μ*M, respectively. Before the experiment, the suspension of 293T cells was incubated in the dark at 37°C for 15 min with PI and FDA. Each experiment was repeated three times, and the fluorescence images were processed using ImageJ software. Prior to the experiment, the number of green fluorescent cells was counted as *g*_0_, and the number of red fluorescent cells was *r*_0_. In the presence of ultrasound, the number of green fluorescent cells was counted as *g*_1_, and the number of red fluorescent cells was counted as *r*_1_. The percentage of living cells and acoustic sonoporation efficiency were evaluated using fluorescence imaging, and the formulae were as follows: percentage of living cells (%) = *g*_1_/*g*_0_ × 100%, and sonoporation efficiency (%) = (*r*_1_ − *r*_0_)/*g*_0_ × 100%.

## Data Availability

The original contributions presented in the study are included in the article/Supplementary Material, further inquiries can be directed to the corresponding author.
