# Supplementary figures and images for: Three-dimensional array of microbubbles sonoporation of cells in microfluidics
Source: Front Bioeng Biotechnol. 2024 Feb 14;12:1353333. doi: 10.3389/fbioe.2024.1353333 (PMC10899490; doi:10.3389/fbioe.2024.1353333)

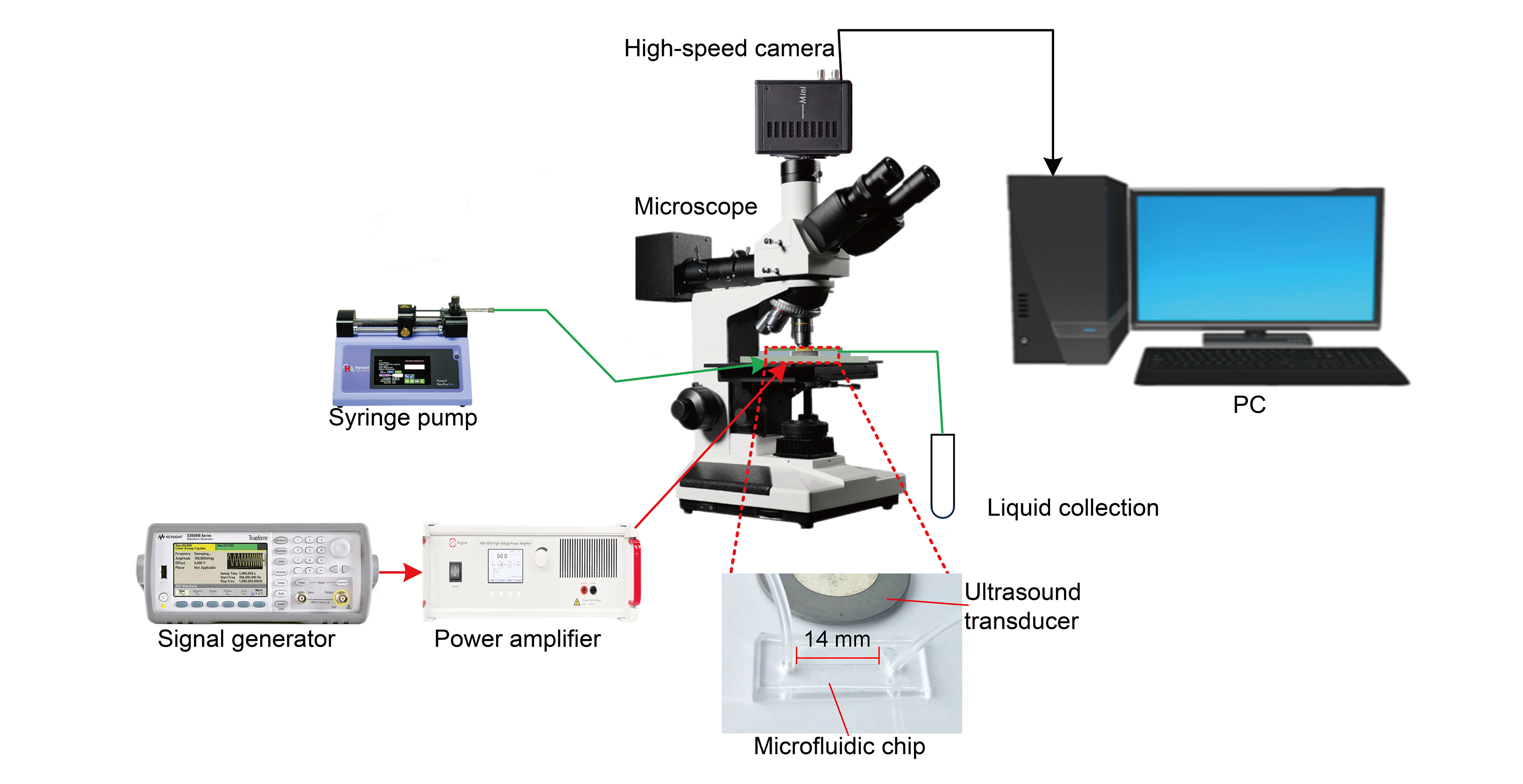

Supplement: Supplementary file 3 [file Image1.JPEG]

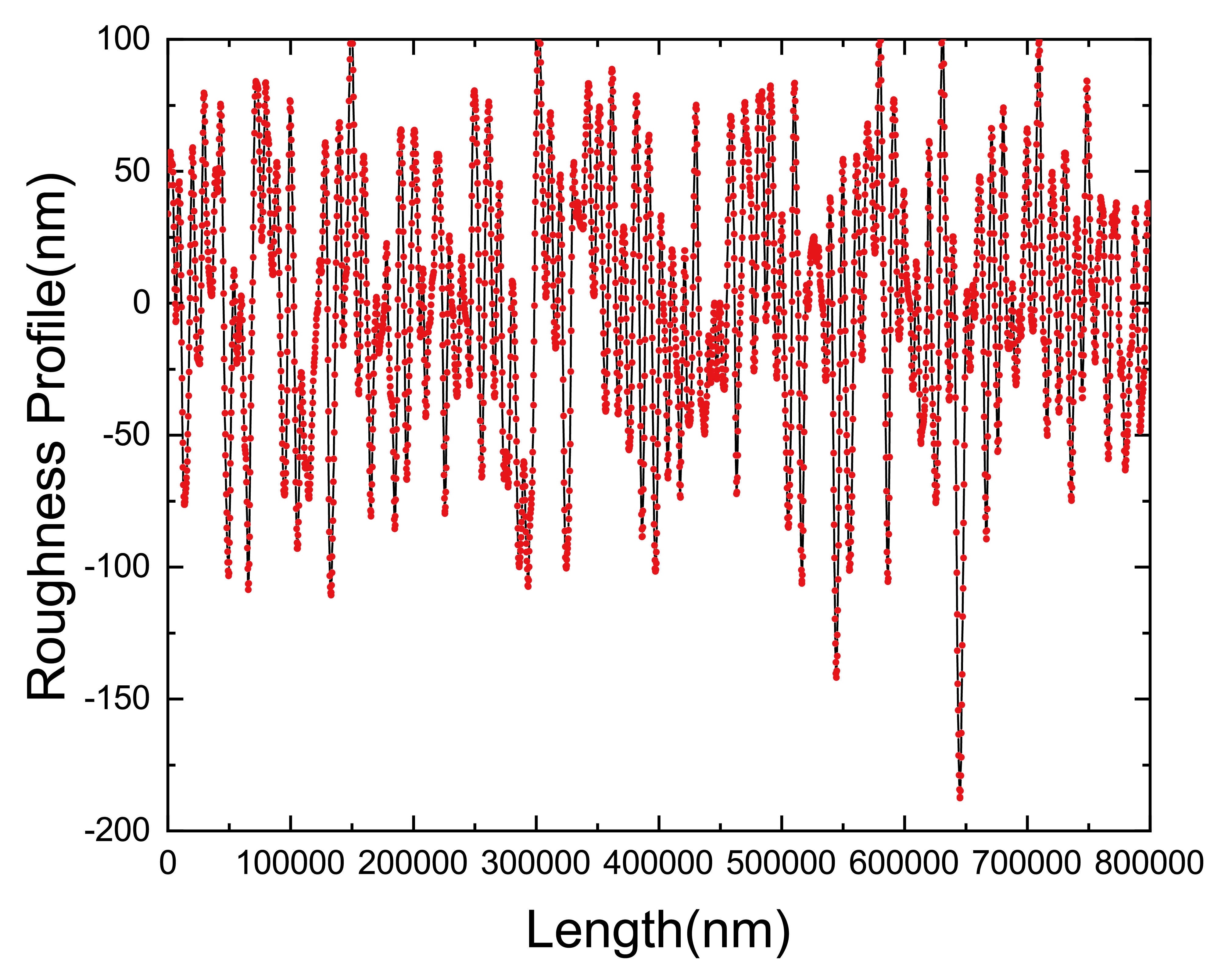

Supplement: Supplementary file 4 [file Image2.JPEG]
