# Supplementary material for: Three-dimensional array of microbubbles sonoporation of cells in microfluidics
Source: Front Bioeng Biotechnol. 2024 Feb 14;12:1353333. doi: 10.3389/fbioe.2024.1353333 (PMC10899490; doi:10.3389/fbioe.2024.1353333)
Supplement: Supplementary file 9 [file DataSheet1.docx]

This PDF file includes:

Figures S1 to S2

Notes S1 to S2

Other supplementary materials for this manuscript include the following:

Movie S1: Fluid flows through the microcavity forming microbubbles.

Movie S2: The motion of the particles as seen from the top view. (three-dimensional microbubbles)

Movie S3: The motion of the particles as seen from the axonometric view. (three-dimensional microbubbles)

Movie S4: The motion of the particles as seen from the axonometric view. (without top microbubbles)

Movie S5: Shape of microbubbles with ultrasound drive.

Movie S6: The captured cells rotated and clustered around the microbubbles.

Movie S7: A brief pause allows the cells to mix more evenly.


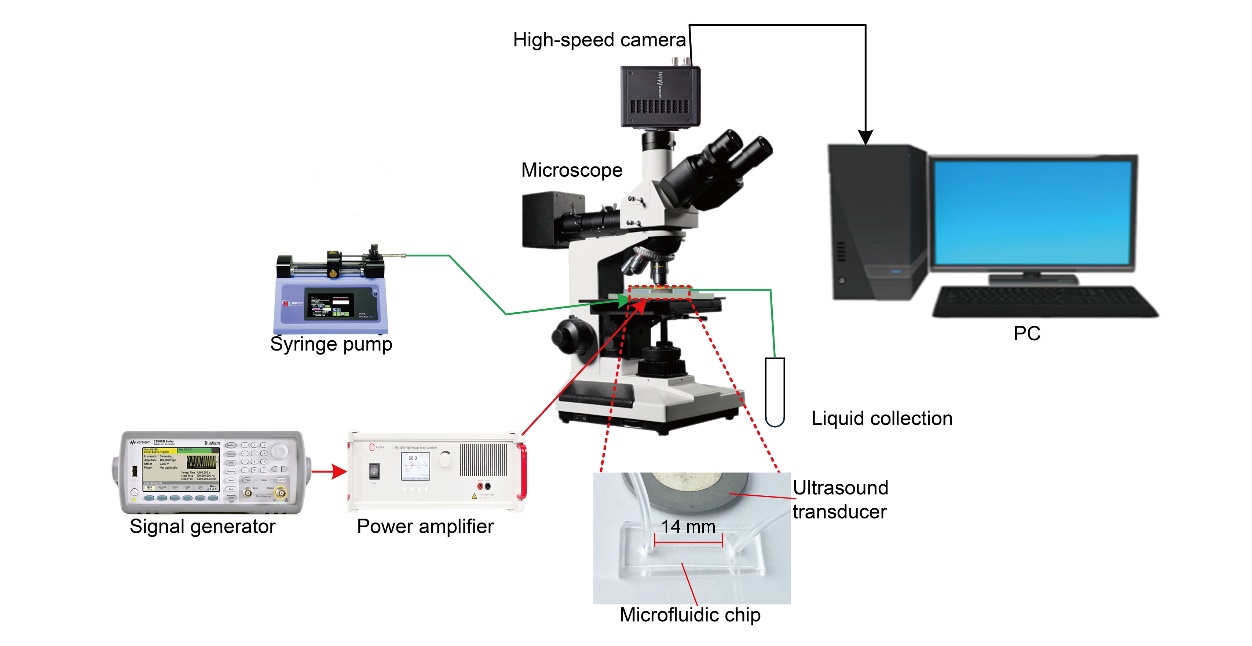


**Fig.S1 Schematic diagram of the experimental setup**


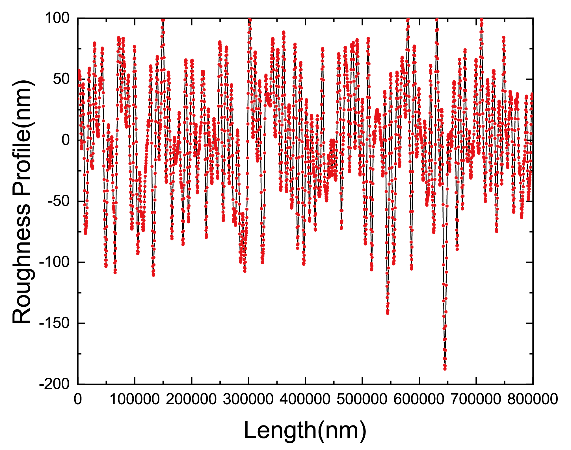


**Fig.S2 Roughness profile of photosensitive resin mold**

**Note S1. The resonance frequency of small amplitude for a microbubble**

The resonance frequency of a trapped microbubble in stationary fluid is estimated by the Rayleigh– Plesset equation.

$f_{t}=\frac{1}{2\pi R_{0}\sqrt{\rho}}\sqrt{3k\left( p+\frac{2\sigma}{R_{0}} \right)-\frac{2\sigma}{R_{0}}}$ (1)

where $\rho$ is the density of the phosphate buffer saline (PBS) solution, $\sigma$ is the surface tension of the PBS solution, $k$ is the polytropic exponent for a bubble containing air, $p$ is the static pressure, and $R_{0}$ is the radius of the microbubble. The frequency of microbubble is calculated to be 95.1 kHz by using Equation (1) ($\rho$=1148 kg m^-3^, $\sigma$=0.064Nm^-1^). $k$ =1.4, $p$ =100kPa, $R_{0}$=32.5 μm).

**Note S2. Theoretical value of shear stress on the trapped cell**

During the process of cell sonoporation, cells are captured and trapped on the surface of microbubbles, and shear stresses near the bubble can be given as follows:

$S=2\pi^{3/2}\varepsilon^{2}\left( \rho{f_{t}}^{3}\mu\right)^{1/2}/R_{0}$ (2)

Where $\rho$ is the density of the phosphate buffer saline (PBS) solution, $\varepsilon$ is the oscillating amplitude of the microbubble, $\mu$ is the dynamic viscosity of the fluid, $f_{t}$ is oscillation frequency,

$R_{0}$ is the radius of the microbubble. According to this Equation (2), the shear stress on the cell is about 455 Pa ($\rho$=1148 kg m^-3^,$\varepsilon$=6.5μm, $f_{t}$=95.1 kHz, $\mu$=1.01*10^-3^pa.s, $R_{0}$=32.5 μm).
